# Real world prevalence of pelvic lymph node involvement in prostate cancer in Asia: do we need a rethink on normograms?

**DOI:** 10.3389/fonc.2025.1583806

**Published:** 2025-05-27

**Authors:** Rachel Lau, Han Jie Lee, Khi Yung Fong, Alvin Yuanming Lee, Yu Guang Tan, Yan Mee Law, Nye Thane Ngo, Jeffrey Tuan, Kae Jack Tay, Lui Shiong Lee, Christopher Cheng, Henry Ho, John Yuen, Kenneth Chen

**Affiliations:** ^1^ Department of Urology, Singapore General Hospital, Singapore, Singapore; ^2^ Department of Diagnostic Radiology, Singapore General Hospital, Singapore, Singapore; ^3^ Department of Pathology, Singapore General Hospital, Singapore, Singapore; ^4^ Division of Radiation Oncology, National Cancer Centre Singapore, Singapore, Singapore; ^5^ Department of Urology, Sengkang General Hospital, Singapore, Singapore

**Keywords:** prostate cancer, nomograms, lymph node involvement, normograms, radical prostatectomy, lymph node staging, pelvic lymph node dissection, Briganti nomogram

## Abstract

**Introduction:**

The Briganti 2019 nomogram stratifies risk of lymph node involvement (LNI) in prostate cancer, reducing unnecessary pelvic lymph node dissection (PLND) during radical prostatectomy (RP). However the applicability of the nomogram in diverse populations remains under-explored, with only one external validation study performed in an Asian population to date. We aim to evaluate the performance of the nomogram in a large tertiary Asian institution.

**Methods:**

A retrospective cohort study was conducted, with analysis of the cancer registry in our tertiary institution of all patients who underwent RP with PLND between 1988 and 2023. The Briganti 2019 nomogram score was retrospectively calculated for each patient, and post-operative data was analyzed to determine rates of LNI in order to determine the performance of the nomogram in our cohort.

**Results:**

437 patients were included, with a median Briganti score of 11.2% (IQR 3.9–28.5%). The mean number of lymph nodes excised per patient was 15.1±12. 292 (66.8%) patients had a Briganti score greater than 7%, but only 8.6% were noted to harbor pN1 disease after RP. In our Asian cohort, the 2019 Briganti nomogram only had a moderate discriminatory ability with an area under the receiver operating characteristic curve (AUC) of 0.77. On multivariate analysis, independent predictors of LNI in our population included percentage of positive biopsy cores [Odds Ratio (OR) 1.02, 95%CI 1.01–1.04, p=0.01] and extraprostatic extension on MRI prostate (OR 3.00, 95%CI 1.20–7.56, p=0.02).

**Conclusion:**

The Briganti 2019 nomogram, while effective in many settings, only had a moderate ability to identify patients with pN1 disease in our Asian cohort. With potential limitations in its generalizability to multiple populations, a re-evaluation of its thresholds and further calibration to other populations might be required.

## 1 Introduction

Globally, prostate cancer (PCa) lies among the top cancers diagnosed in men worldwide and the fifth leading cause of cancer death (1), and its prevalence and characteristics varies across different populations. Lymph node involvement (LNI) is a key factor for prognostication in PCa, and pelvic lymph node dissection (PLND) has been recommended for nodal staging in high-risk or selected intermediate-risk localized PCa (2). However, given the increased operative risks associated with PLND, current guidelines suggest the usage of nomograms for risk stratification of intermediate-risk patients prior to offering it (3).

Previously, the Briganti 2012, Briganti 2017, and the Memorial Sloan Kettering Cancer Center (MSKCC) nomograms have utilized clinical and pathological variables to aid in risk stratification for the risk of LNI (4–7). The latest iteration of the Briganti nomogram was released in 2019, and was based on multi-parametric MRI prostate findings and MRI-guided targeted biopsies for the risk stratification of patients, a feature which was unique to this nomogram. Accepting a threshold of 7% would spare 56% of unnecessary PLNDs, at the expense of missing 1.6% of patients with LNI (8) – and this threshold has since been accepted into international guidelines.

However, the main concern with nomograms is that they tend to be population-specific, and the generalizability of such nomograms in other diverse populations might be uncertain (9), especially since differences in the genetic makeup and environment of a population can significantly influence the prevalence, characteristics, and natural history of a disease (10). The Briganti 2019 nomogram was developed based on data from European institutions, where the majority of patients included were Caucasians; the majority of studies validating it were also inherently European in nature. Its applicability in diverse populations therefore remains under-explored, with only one study to date exploring its application in an Asian population (11). Our study therefore aims to evaluate the performance of the Briganti 2019 nomogram in a large tertiary Asian institution.

## 2 Methods

A retrospective cohort study was conducted at our tertiary institution, evaluating patients who underwent radical prostatectomy (RP) and PLND between 1988 and 2023. We included patients with localized PCa diagnosed with MRI prostate and an MRI-targeted biopsy, and subsequently underwent RP and PLND. Patients with incomplete data precluding calculation of the Briganti 2019 score were excluded from our study.

Data collected from our prospectively-maintained cancer registry included demographic information, pre-operative PSA levels, clinical stage, Gleason score (GS) on MRI-targeted biopsy, MRI lesion diameter, percentage of biopsy cores with clinically significant PCa at systematic biopsy, histological LNI, and postoperative outcomes. Biochemical recurrence (BCR) was defined as PSA ≥0.2 ng/mL with a second confirmatory level > 0.2 ng/mL after RP. The Briganti 2019 score was calculated for each patient based on these parameters. Patients were classified according to EAU risk classification for localized PCa, pathological nodal status (pN0 vs pN1), and Briganti 2019 score (above or below 7% risk threshold).

Excel version 16.87 and RStudio version 2024.04.2 + 764 were used for statistical analysis. The Mann-Whitney U and Chi-square tests were used to evaluate patient characteristics, while multivariate logistic regression was used to determine variables that significantly predicted post-operative LNI. The Area under the receiver-operating characteristic curve (AUC) was calculated to evaluate the discriminatory performance of the Briganti 2019 model in our cohort. Statistical significance was set at p<0.05.

## 3 Results

### 3.1 Patient baseline characteristics

A total of 437 patients were included in this study, and all of them underwent robotic-assisted RP with PLND. Their characteristics are summarized in **Table 1**. Median age was 67 (62.8–70.7) years, median pre-operative PSA was 9.1 (5.4–14.1) ng/mL, and most patients had intermediate-risk disease (n = 314, 71.9%) (3). Mean operation time and post-operative hospital stay was 245±51 minutes 2.86±1.65 days respectively. Median post-operative follow-up was 20.7 (0.1–67.0) months.

**Table 1:** Baseline patient characteristics.

Patient and disease characteristics	Overall	pN0	pN1	P value
Median age, years (IQR)	67.0 (62.8 – 70.7)	66.9 (62.8 – 70.5)	69.3 (64.0 – 73.6)	0.08
Median prostate volume, mL (IQR)	35.1 (26.7 – 46.0)	35.1 (26.0 – 46)	35.3 (32.2 – 43.5)	0.65
Median follow up period, days (IQR)	490 (320 – 879)	478 (315.5 – 870.5)	538 (451.5 – 1177)	<0.05
Median Briganti 2019 score, % (IQR)	11.2 (3.9 – 28.5)	10.8 (3.6 – 25.0)	37.9 (18.0 – 56.8)	<0.05
Median preoperative PSA, ng/mL (IQR)	9.1 (6.5 – 14.1)	9.0 (6.4 – 13.9)	12.9 (8.3 – 21)	<0.05
Median MRI lesion diameter, mm (IQR)	15 (11.0 -20.0)	15 (11.0 – 19.8)	20.0 (16.5 – 240.0)	<0.05
Median percentage of positive biopsy cores, % (IQR)	27.3 (16.0 – 43.8)	26.6 (15.7 – 42.1)	50.0 (25.4 – 68.5)	<0.05
Patients with seminal vesical involvement on MRI prostate, n (%)	29 (6.6)	24 (5.9)	5 (18.5)	<0.05
Patients with extraprostatic extension on MRI prostate, n (%)	106 (24.3)	94 (22.9)	12 (44.4)	<0.05
Gleason Grade group, n (%)				
Grade group 1	31 (7.1)	30 (7.3)	1 (3.7)	<0.05
Grade group 2	160 (36.6)	153 (37.3)	7 (25.9)	
Grade group 3	106 (24.3)	100 (24.4)	6 (22.2)	
Grade group 4	107 (24.5)	103 (25.1)	4 (14.8)	
Grade group 5	33 (7.6)	24 (5.9)	9 (33.3)	
Patients with Gleason grade group 4-5, n (%)	140 (32.0)	127 (31.0)	13 (48.1)	0.06
EAU risk stratification, n (%)				
Low risk	18 (4.1)	18 (4.4)	0 (0)	0.32
Intermediate risk	314 (71.9)	296 (72.2)	18 (66.7)	
High risk	105 (24.0)	96 (23.4)	9 (33.3)	
Pathological stage, n (%)				
pT2	244 (55.8)	242 (59.0)	2 (7.4)	< 0.05
pT3a	126 (28.8)	117 (28.5)	9 (33.3)	
pT3b/pT4	67 (15.3)	51 (12.4)	16 (59.3)	
Biochemical recurrence, n (%)	53 (13.1%*)	47 (12.2%^#^)	6 (31.6%^)	<0.05
Median days to biochemical recurrence, days (IQR)	440 (262.3 – 699.3)	466 (276.8 – 747.8)	245 (90.8 – 369.3)	0.06

*Data only available for 404 of 437 patients.

^#^Data only available for 385 of 410 patients.

^Data only available for 19 of 27 patients.

Of the 437 patients, 27 had LNI (pN1) while 410 did not (pN0). Within our series, the mean number of lymph nodes(LN) dissected was 15.1±12.0. There was no significant difference between the mean number of LNs excised across patient who underwent RP and PLND from 2007–2014 compared to those from 2015 - 2023 (14.1 vs 15.1, p = 0.75). Notably, the pN1 cohort had a higher proportion of patients with high-risk disease compared to the pN0 cohort (33.3% vs 23.4%), as well as higher pre-operative PSA (12.9 vs 9.0), median MRI lesion diameter (20mm vs 15mm), and proportion of patients with MRI-defined extraprostatic extension (44.4% vs 22.9%) and seminal vesicle invasion (18.5 vs 5.9%).12.2% of patients in the pN0 cohort experienced biochemical recurrence compared to 31.6% of patients with pN1 disease. Biochemical recurrence occurred earlier in the pN1 cohort at a median of 245 (90.8-369.3) days compared to the pN0 cohort with a median of 466 (276.8 – 747.8) days, but there was not statistically significant.

The cohort had an overall median Briganti 2019 score of 11.2% (3.9 – 28.5%). The pN0 cohort had a significantly lower median Briganti score of 10.8% (3.6–25.0%) compared to the pN1 cohort 37.9% (18.0–56.8%). Most patients included in our present analysis had a Briganti score greater than 7% (N=292; 66.8%), but LNI was only noted in 8.6% (N=25).

### 3.2 Multivariate analysis

A multivariate analysis was performed to determine whether each individual clinical parameter included in the Briganti 2019 nomogram were independent predictors for LNI in our cohort (**Table 2**). The percentage of positive biopsy cores and the presence of extra-prostatic extension on MRI prostate were independent predictors for LNI, with an odds ratio (OR) of 1.02 (95%CI 1.01–1.04, p=0.01) and 3.00 (95%CI 1.20–7.56), p=0.02) respectively. The remaining clinical parameters including PSA, Gleason group 4-5, MRI lesion diameter, and seminal vesical involvement on MRI prostate were not predictors for LNI in our cohort.

**Table 2:** Multivariate logistic regression analysis assessing the prediction of LNI based on Briganti 2019 nomogram clinical parameters.

Clinico-pathological parameters	Coefficient	Odds Ratio	95% CI	P-value
Preoperative PSA	0.0021	1.00	0.98 - 1.02	0.82
Percentage of positive biopsy cores	0.0237	1.02	1.01 - 1.04	0.01
Gleason Group 4-5	0.5059	1.66	0.72 - 3.77	0.23
MRI lesion diameter	0.0184	1.02	0.96 - 1.07	0.51
Extraprostatic extension on MRI prostate	1.0977	3.00	1.20 - 7.65	0.02
Seminal vesical involvement on MRI prostate	0.9640	2.62	0.62 - 9.93	0.17

R^2^: 0.13, p < 0.05.

### 3.3 Performance characteristics of the Briganti 2019 nomogram

At the proposed Briganti 2019 nomogram threshold of 7%, there were 292 patients (66.8%) with a score greater than 7%, where 25 patients (8.6%) had LNI while 267 (91.4%) did not. 145 patients (33.2%) had a calculated Briganti 2019 score below 7%, and 2 patients (1.4%) had LNI, while the 143 (98.6%) did not. If the threshold of 7% was similarly adhered to in our Asian cohort, we would only spare 33.2% of patients an unnecessary PLND at the expense of missing 1.4% of patients with LN disease. Sensitivity, Specificity, and negative predictive value (NPV) for identification of LNI at a threshold of 7% was 92.59%, 34.88%, and 98.62% respectively (32).

When changing the threshold for LNI to 8%, the specificity improves slightly from 34.88% to 39.02%, with minimal impact on sensitivity (92.59%) and NPV (98.62%). Beyond an 8% cut off, sensitivity begins to drop significantly with a greater number of LNI being missed (**Table 3**).

**Table 3:** Prevalence of lymph node involvement at adjusted Briganti 2019 score cut offs.

Briganti 2019	Below cut off			Above cut off			Specificity (%)	Sensitivity (%)	NPV (%)
Cut off	Total	Without LNI	With LNI	Total	Without LNI	With LNI			
2%	13 (2.97%)	13	0	424 (97.03%)	397	27	3.17	100.00	100.00
3%	78 (17.85%)	77	1	359 (82.15%)	333	26	18.78	96.30	98.72
4%	116 (26.54%)	115	1	321 (73.46%)	295	26	28.05	96.30	99.14
5%	126 (28.83%)	125	1	311 (71.17%)	285	26	30.49	96.30	99.21
6%	135 (30.89%)	133	2	302 (69.11%)	277	25	32.44	92.59	98.52
7%	145 (33.18%)	143	2	292 (66.82%)	267	25	34.88	92.59	98.62
8%	162 (37.07%)	160	2	275 (62.93%)	250	25	39.02	92.59	98.77
9%	179 (40.96%)	176	3	258 (59.04%)	234	24	42.93	88.89	98.32
10%	189 (43.25%)	186	3	248 (56.75%)	224	24	45.37	88.89	98.41
11%	216 (49.43%)	211	5	221 (50.57%)	199	22	51.46	81.48	97.69
12%	229 (52.40%)	224	5	213 (48.74%)	191	22	54.63	81.48	97.82
13%	241 (55.15%)	236	5	196 (44.85%)	174	22	57.56	81.48	97.93
14%	252 (57.67%)	247	5	185 (42.33%)	163	22	60.24	81.48	98.02
15%	258 (59.04%)	253	5	179 (40.96%)	157	22	61.71	81.48	98.06
20%	299 (68.42%)	291	8	138 (31.58%)	119	19	70.98	70.37	97.32
25%	315 (72.08%)	307	8	122 (27.92%)	103	19	74.88	70.37	97.46
30%	333 (76.20%)	321	12	104 (23.80%)	89	15	78.29	55.56	96.40
35%	350 (80.09%)	337	13	87 (19.91%)	73	14	82.20	51.85	96.29
40%	370 (84.67%)	356	14	67 (15.33%)	54	13	86.83	48.15	96.22
50%	394 (90.16%)	377	17	43 (9.84%)	33	10	91.95	37.04	95.69
60%	409 (93.59%)	389	20	28 (6.41%)	21	7	94.88	25.93	95.11
70%	419 (95.88%)	394	25	18 (4.12%)	16	2	96.10	7.41	94.03
80%	426 (97.48%)	401	25	11 (2.52%)	9	2	97.80	7.41	94.13
90%	430 (98.40%)	404	26	7 (1.61%)	6	1	98.54	3.70	93.95
100%	437 (100.00%)	410	27	0 (0.00%)	0	0	100.00	0.00	93.82

**Figure 1:**
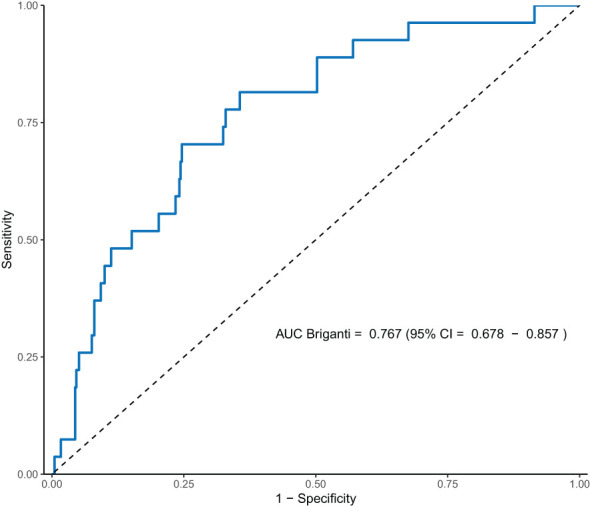
ROC curve of Briganti’s 2019 nomogram in our cohort.

A Receiver-Operating Characteristic (ROC) curve was plotted based on the performance of the Briganti 2019 model in our cohort (**Figure 1**), and the calculated AUC was 0.77 (95% CI 0.68–0.86). The threshold at which AUC is maximized is at 15.8%, with a sensitivity of 81.5% and a specificity of 64.4%.

We also evaluated the performance of the Briganti 2019 across different cohorts, presented in **Table 4**, by incorporating 2 additional external validations performed to the table initially presented by Fukagawa et al, and compared it to the AUC derived from our cohort. Remarkably, the Briganti 2019 nomogram had a poorer discriminatory performance in our cohort and Fukugawa’s Japanese cohort (AUC 0.71), compared to the original population of patients and most other western external validation cohorts.

**Table 4:** AUCs of the Briganti 2019 nomogram in the original and external validation cohorts.

Normogram	Original cohort (8)	Our cohort	Fukagawa et al. (11)	Gandaglia et al. (13)	Diamond et al. (12)	Malkiewicz et al. (14)	Frego et al. (15)	Soeterik et al. (16)
Briganti 2019	0.86	0.77	0.71	0.79	0.80	0.80	0.82	0.79

When comparing baseline characteristics, our cohort is considered higher risk pre-operatively for LNI across most parameters included in the nomogram compared to Briganti’s original cohort (**Table 5**). Of note, our pN0 cohort had greater high-risk features in all parameters except for percentage of positive biopsy cores, compared to Briganti’s cohort, similar to Fukagawa’s findings.

**Table 5:** Comparing baseline patient characteristics across different cohorts.

Baseline patient characteristic	External validation cohort (n = 437)			Briganti’s original cohort (n = 497)			Fukagawa et al. (n = 278)		
	pN0	pN1	P value	pN0	pN1	P value	pN0	pN1	P value
Patients	410 (93.6%)	27 (6.2%)		435 (87.5%)	62 (12.5%)		259 (93.2%)	19 (6.8%)	
Median preoperative PSA	9.0	12.9	<0.05	7.2	11.0	<0.05	8.0	11.4	<0.05
Percentage of patients with Gleason grade group 4-5 (%)	31.0	48.1	0.06	16.0	48.0	<0.05	46.0	63.0	<0.05
Median MRI lesion diameter (mm)	15	20	<0.05	10	15	<0.05	10	13	0.16
Median percentage of positive biopsy cores (%)	26.6	50.0	<0.05	33.0	55.0	<0.05	38.0	56.0	<0.05
Percentage of patients with extraprostatic extension on MRI prostate (%)	22.9	44.4	<0.05	12.0	31.0	<0.05	23	37	0.38
Percentage of patients with seminal vesical involvement on MRI prostate (%)	5.9	18.5	<0.05	3.0	22.0	<0.05	1.9	0	

## 4 Discussion

### 4.1 Comparing external validation studies of the Briganti 2019 nomogram

The Briganti 2019 nomogram has been externally validated by multiple studies in primarily Caucasian populations, with findings consistent with Briganti’s own observations (12–16). However, the validity of Briganti’s model remains understudied in non-European populations, with only one study to date examining the nomogram’s validity in a primarily Asian population (11).

At the proposed 7% cut off, given the high Briganti 2019 scores of our patients, the number of unnecessary PLNDs that we would spare is low compared to Briganti’s original cohort. Even as we increase the cut off, the number of unnecessary PLNDs spared remains lower than Briganti’s observations with minimal change in sensitivity, specificity, and negative predictive value (**Table 3**). These findings are similar to Fukagawa’s observations, where they report a relatively higher score in their validation cohort compared to Briganti’s original cohort, yet with lower rates of LNI suggesting that the pre-operative evaluation of their cohort tended to be overestimated (11).

When evaluating the AUC of the Briganti 2019 nomogram in various populations, Fukagawa et al. found that the AUC in their own Japan-based cohort was lower compared to the three other existing external validation reports at time of publication, which were all based on a European cohort (11). Although the model did perform better in our cohort compared to Fukagawa’s cohort, we found that our AUC was lower compared to the AUC calculated in the original and other external validation cohorts (**Table 4**). This suggests a possible poorer fit of the model in the Asian population.

### 4.2 Prostate cancer characteristics amongst different demographics

This difference in patient characteristics between our cohort and Briganti’s original cohort could account for the poor fit of the nomogram in predicting LNI in our cohort compared to other external validation studies. Our multivariate logistic regression analysis showed that many of the clinical parameters included in the nomogram were not significant independent predictors for LNI. The only two parameters that were significant independent predictors were percentage of positive biopsy cores on MRI targeted prostate biopsy (OR 1.02, 95% CI 1.01 – 1.04, p = 0.01), and extraprostatic extension on MRI prostate (OR 3.00, 95% CI 1.20 – 7.56, p = 0.02). This suggests a possible difference in not only disease characteristics, but also in progression and tumor biology.

Differences in PCa characteristics and disease progression has been observed across various ethnic groups. Notably, Asian men have been observed to have more favorable survival rates despite poorer prognostic profiles (17). There have been various theories suggesting reasons for the discordance of disease progression between racial groups. Endogenous testosterone level has been shown to increase PCa risk, and in the presence of PCa, has been found to correlate with increased risk of disease progression (18). Asian men have been found to have lower testosterone levels compared with other racial groups, with a suggested role of genetics and lifestyle factors, such as dietary intake (19–21). Furthermore, lifestyle affecting intraprostatic microbiome and gut microbiome has potential effects on the risk and progression of PCa (22). Tumor genomics have also shown variability across different racial groups, and likely contributes to differences in disease characteristics and progression in various racial groups (23).

It is important to acknowledge, however, that with increased globalization and continuous shifts in sociodemographic and cultural trends, extrinsic factors such as environmental or lifestyle factors are dynamic and may lead to future changes in the observed patterns of disease progression (24). Other factors to consider include variations in access to healthcare, screening practices, and common surgical procedures across different countries. Therefore, although some countries may have similar racial profiles, differences in practice may add to differences in disease profile and risk of progression across different populations (25).

Given the differences in tumor characteristics and disease progression between populations, existing nomograms are limited to the populations in which they are created for, and we should approach the application of these nomograms to other population groups with caution. Moving forward, creating population specific nomograms will allow for more precise risk stratification, to aid decision making (9). Furthermore, the incorporation of new imaging modalities such as PSMA PET/CT and genetic biomarkers will allow for better characterization of disease and further improve our predictive tools (26–29).

### 4.3 Limitations

There are a few limitations of this study to note. Firstly, given that it is not routine practice for all patients with PCa to undergo PLND, there is a selection bias within our patient cohort in which patients who are higher risk for LN disease are more likely to be offered PLND. Therefore amongst low risk patients who are less likely to have undergone PLND, there are potentially patients with missed nodal disease.

Secondly, MRI reporting and MRI targeted biopsy techniques are subjective and clinician dependent. In Briganti’s cohort, although clinicians were advised to take a minimum of 2 targeted cores for each suspicious lesion and at least 6 random cores outside the MRI-targeted biopsy area, the final decision for number of targeted and systematic cores taken was still dependent on the judgement of each treating physician (8). Within our institution, there is no standardized practice for number of cores to take in a MRI-targeted biopsy, which could potentially affect rates of false negatives. Furthermore, reporting of multiparametric MRI prostate is reporter-dependent, and can vary between institutions.

Lastly, given the extended time frame of our data, there is a lack of data regarding PLND templates and surgical technique. Although the mean number of nodes dissected (n = 15.1) is comparable with Briganti’s’ original cohort, suggesting adequate dissection, we are unable to comment on the extent of PLND in our patient cohort. We therefore have to interpret our data with caution, recognizing the challenge of direct application of our findings on other institutions due to possible differences in practice.

## 5 Conclusion

The Briganti 2019 nomogram, while effective in many settings, shows poorer performance in our PCa Asian cohort with high-risk features, necessitating a re-evaluation of its thresholds and clinical parameters included in the model. Population-specific adjustments and the incorporation of additional predictive factors may improve the predictive tools of LNI and further improve our selection of patients with PLND.

## Data Availability

The raw data supporting the conclusions of this article will be made available by the authors, without undue reservation.
